# Inhibiting α-Synuclein Oligomerization by Stable Cell-Penetrating β-Synuclein Fragments Recovers Phenotype of Parkinson's Disease Model Flies

**DOI:** 10.1371/journal.pone.0013863

**Published:** 2010-11-10

**Authors:** Ronit Shaltiel-Karyo, Moran Frenkel-Pinter, Nirit Egoz-Matia, Anat Frydman-Marom, Deborah E. Shalev, Daniel Segal, Ehud Gazit

**Affiliations:** 1 Department of Molecular Microbiology and Biotechnology, Tel Aviv University, Tel Aviv, Israel; 2 Wolfson Centre for Applied Structural Biology, Hebrew University of Jerusalem, Jerusalem, Israel; National Institutes of Health, United States of America

## Abstract

The intracellular oligomerization of α-synuclein is associated with Parkinson's disease and appears to be an important target for disease-modifying treatment. Yet, to date, there is no specific inhibitor for this aggregation process. Using unbiased systematic peptide array analysis, we indentified molecular interaction domains within the β-synuclein polypeptide that specifically binds α-synuclein. Adding such peptide fragments to α-synuclein significantly reduced both amyloid fibrils and soluble oligomer formation *in vitro*. A retro-inverso analogue of the best peptide inhibitor was designed to develop the identified molecular recognition module into a drug candidate. While this peptide shows indistinguishable activity as compared to the native peptide, it is stable in mouse serum and penetrates α-synuclein over-expressing cells. The interaction interface between the D-amino acid peptide and α-synuclein was mapped by Nuclear Magnetic Resonance spectroscopy. Finally, administering the retro-inverso peptide to a *Drosophila* model expressing mutant A53T α-synuclein in the nervous system, resulted in a significant recovery of the behavioral abnormalities of the treated flies and in a significant reduction in α-synuclein accumulation in the brains of the flies. The engineered retro-inverso peptide can serve as a lead for developing a novel class of therapeutic agents to treat Parkinson's disease.

## Introduction

The role of protein oligomerization and deposition in neurodegenerative diseases has become evident in diverse disorders including Alzheimer's disease (AD), Huntington's disease, Parkinson's disease (PD), Creutzfeldt-Jacob disease, prion disease, and type II diabetes [1]–[8]. In each case of these diseases, a different endogenous protein self-assembles into highly ordered fibrillar structures. Though there is no specific sequence homology among these proteins, they are all thought to involve important conformational changes in the corresponding protein, usually the production of β-sheet structures which have a strong tendency to aggregate into water-insoluble fibrous polymers [5], [9]. It is currently debated whether the precipitated insoluble fibrils or actually soluble oligomers are the cytotoxic aggregative elements that are associated with the pathologies of each of these diseases [1], [10]–[16]. Moreover, it is also not yet clear whether the soluble oligomers are formed in an on-pathway fashion as intermediates eventually leading to the non-soluble fibrils and plaques or formed off- pathway by a separate mechanism.

PD, the second most common form of neurodegenerative diseases after AD, is a devastating neurological disease without cure, affecting 1–2% of the elderly population. It is characterize by loss of neuromelanin-containing dopaminergic neurons in the *substantia nigra pars compacta* with presence of eosinophillic, intracytoplamic, proteinaceous inclusions termed Lewy bodies (LB) and dystrophic Lewy neuritis (LN) in surviving neurons. Among the clinical features of PD are motor impairments involving resting tremor, bradykinesia, postural instability and rigidity along with non-motoric symptoms such as autonomic, cognitive and psychiatric problems. The cause of these pathological characteristics is not yet fully understood but it is believed that environmental factors as well as a genetic causation or a combination of the two might result in the abovementioned clinical syndromes [17]. It is now known that less than 10% of the PD cases have a strict familial etiology while the majority of cases are sporadic [18]. Among the mutations associated with familial PD, three missense mutations in the α-synuclein (α-syn) gene termed A53T, A30P and E46K have been widely characterized [19], [20].

The 140 amino acid α-syn protein is a small, highly charged, natively unfolded protein, first identified in 1977 as the major component of LB and LN [21], [22]. This was followed by the discovery of the point mutations in its gene [23]. Besides its role in PD, the protein is associated with pathological inclusions of several other neurodegenerative diseases including dementia associated with Lewy bodies, LB variant of AD, and multiple system atrophy [24]. Three major regions are recognized in α-syn: the amino terminal region containing several imperfect repeats of the KTKEGV sequence, a hydrophobic central domain called the non-amyloid component (NAC) region, and the carboxy terminal region characterized by its highly negatively charged amino acids [25], [26]. The α-syn protein is predominantly expressed in neurons of the central nervous system (CNS), where it is localized at pre-synaptic termini in close proximity to synaptic vesicles, and can associate with lipid membranes by forming amphipathic α-helices [24].

α-syn is a member of the family of synuclein proteins, along with β-synuclein (β-syn) and γ-synuclein (γ-syn) [27]. α-syn and β-syn are found primarily in brain tissues located mainly in the pre-synaptic nerve terminals, while γ-syn is found primarily in the peripheral nervous system and the retina, although it has also been observed to be highly expressed in some tumor tissues, including breast, ovarian and bladder tissues [24].

The sequence of the three synuclein protein is highly conserved, especially within their N terminal domains. When the sequences of α-syn and β-syn are compared, a major difference within the hydrophobic central domain is apparent: β-syn, a 134 amino acid protein, lacks the NAC region of α-syn and, in contrast to the latter, does not aggregate to form amyloid fibrils under various stress conditions such as free radicals or increased concentration [9], [28]. Moreover, β-syn was shown to inhibit the aggregation of α-syn *in vitro* in a dose-dependent manner [29], [30]. Uversky et al. proposed that this inhibition occurs due to the incorporation of β-syn into oligomeric intermediates which in turn leads to their stabilization. It is hypothesized that β-syn interacts with a putative transient oligomeric intermediate of α-syn, normally on its way to form fibrils, resulting in the stabilization of the α-syn intermediate and blocking its conversion into fibrils [31].

Based on the finding that β-syn inhibits the aggregation of α-syn, Windisch and colleagues further examined the inhibitory potential of several deletion mutants of β-syn, focusing on the N-terminal 1–15 amino acids of the protein. The researchers generated a peptide library containing different variations of amino acid composition derived from this specific sequence of β-syn, with the aim of finding a peptide that could be used for therapeutic applications, or could serve as a basis for developing peptidomimetic small molecules [32].

In the present work, we systematically mapped the entire sequence of β-syn in order to identify other domains that have the potential to facilitate the molecular recognition between β-syn and α-syn. Based on these findings, we rationally designed modified metabolically-stable peptides and analyzed their interaction with α-syn using chemical and biophysical methods. These peptides could be internalized into cultured cells and caused a marked amelioration of the behavioral impairments in a PD fly model.

## Methods

### Peptide array

The molecular mapping of β-syn fragments binding α-syn was performed using peptide array technology. Decamer peptides corresponding to overlapping sequences of the full length, 134 amino acids, β-syn protein were synthesized on a cellulose membrane matrix (JPT Peptide Technologies GmbH, Germany). The first 15 amino acids were synthesized with 9 amino acids overlap with a shift of one amino acid from one peptide to its subsequent. The importance of this region was suggested previously as a binding domain [32]. The rest of the peptide was synthesized with five amino acids overlap. The peptides were covalently bound to a Whatman 50 cellulose support via the C-terminal amino acid residues. Two spots on the membrane contained libraries of non-relevant peptides. These libraries were supplied and synthesized by JPT company and were used as a negative control.

After blocking, the membrane was incubated in the presence of N-terminal histidine-tagged recombinant α-syn, followed by treatment with HRP-conjugated anti-His monoclonal antibody. In order to identify the strongest binding sites, an additional membrane containing β-syn decamer peptides with a 9 amino acid overlap between each sequential peptide along the protein was used under more stringent conditions; this time we used a smaller amount of α-syn reacting with the membrane and half the concentration of the secondary antibody. Once β-syn binding sites were identified, small β-syn derived peptides were synthesized.

### Expression and purification of α-syn

The protein was expressed in pT7-7 BL21 *E. coli* bacteria. For over expression, bacterial cultures were grown to logarithmic stage in the presence of ampicillin (100 mg/l) and protein expression was induced using IPTG (1 mM) for 3 hours. The bacterial pellet was re-suspended in TEN buffer (50 mM Tris pH 8.0, 10 mM EDTA, 150 mM NaCl), and frozen at −80°C until purification.

Protein purification was accomplished using a non-chromatographic method as described by Volles and Lansbury [33]. Briefly, after boiling and centrifugation of the bacterial pellet, the supernatant was removed to a fresh tube and streptomycin sulfate (136 µl of a 10% solution/ml supernatant) and acetic acid (glacial, 228 µl/ml supernatant) were added, followed by additional centrifugation for two minutes. The supernatant was removed again and then precipitated with ammonium sulfate (saturated ammonium sulfate at 4°C was used 1∶1 v∶v with supernatant). The precipitated protein was collected by centrifugation, and the pellet was washed once with 1 ml of 50% ammonium sulfate solution (4°C; 1∶1 v∶v saturated ammonium sulfate (4°C):water). The washed pellet was re-suspended in 900 µl of 100 mM ammonium acetate (to form a cloudy solution) and precipitated by adding an equal volume of 100% ethanol at room temperature. Ethanol precipitation was repeated once more, followed by a final re-suspension in 100 mM ammonium acetate, overnight dialysis to water at 4°C, freezing in liquid nitrogen, and lyophilization.

For validation, the α-syn protein was later purified by an additional method using chromatography and was tested for aggregation inhibition using the same peptides. Briefly, the bacterial pellet was re-suspended in 50 mM Tris, 50 mM KCl, 5 mM MgAc, 0.1% NaN_3_, pH 8.5 and was supplemented with 300 µM PMSF inhibitors. The solution was sonicated and ultracentrifuged for 30 minutes at 14,000 RPM at 4°C. The resulting supernatant was boiled for 15 minutes in a water bath. To separate the precipitated proteins from the solution, the sample was centrifuged for 20 minutes (7,000 RPM at 4°C). The supernatant was filtered through a 0.45 µm filter and was kept at 4°C for further purification.

The filtrated supernatant was applied on a HiPrep 16/10 QFF anion exchange column. At 20 mM Tris, pH 8, α-syn has a net negative charge, and can therefore interact with the positively charged moieties (NH^+^) of the column. The protein was eluted using 30–40% of 20 mM Tris and 1 M NaCl solution (pH 8) and was subjected to a HiLoad 26/20 superdex 200 size exclusion column. Anion exchange fractions were loaded with 50 mM Tris/HCl and 150 mM NaCl buffer (pH 7.5), and the monomer fraction was analyzed by SDS-PAGE and was collected, dialyzed overnight to ddH_2_O at 4°C, frozen in liquid nitrogen, and lyophilized.

### ThT fluorescence assay of fibril formation

α-syn was dissolved to a concentration of 200 µM in 100 mM Tris buffer (pH 7.4). In order to obtain the monomeric fraction, the protein was filtered through a 100 kDa centricon. Since α-syn is not a globular but rather a natively unfolded protein, only monomers and some dimers pass through the membrane. The monomeric protein was immediately mixed with or without different β-syn-derived peptides at a 1∶1 ratio to a final concentration of 100 µM. The samples were incubated at 37°C with agitation of 850 RPM as described by Tsigelny, et al. [34] and the rate of fibrillogenesis was monitored using the thioflavin T (ThT) fluorescence assay (excitation at 450 nm, 2.5 nm slit, and emission at 480 nm, 5 nm slit), after three days of incubation. ThT was added to a 500-fold diluted sample and fluorescence was measured using a Jobin Yvon Horiba Fluoromax 3 fluorometer. To assure that β-syn-derived peptides don't aggregate, their fluorescence was measured as control and subtracted from the test samples.

### Transmission electron microscopy

Samples (10 µl) from the α-syn ThT fluorescence assay (with and without inhibitors) were placed on 400-mesh copper grids covered by carbon-stabilized Formvar film (SPI Supplies, West Chester, PA). After 1.5 minutes, excess fluid was removed, and the grids were negatively stained with 10 µl of 2% uranyl acetate solution for two minutes. Finally, excess fluid was removed and the samples were viewed by a JEOL 1200EX electron microscope operating at 80 kV.

### Determination of soluble oligomer formation

Monomeric α-syn was dissolved to a concentration of 200 µM in 100 mM Tris buffer (pH 7.4) and was immediately mixed with or without different β-syn-derived peptides at a 1∶1 ratio to a final concentration of 100 µM, as was described above for the ThT fluorescence assay. After the samples were agitated at 37°C for several days, 10 µl of the protein were centrifuged at 13,000 RPM for 10 minutes, the supernatant was collected and was electrophoresed in acrylamide gel using native loading buffer without β-mercaptoethanol or boiling. The gel was washed three times in ddH_2_O and samples were transferred to the nitrocellulose membrane using a semi-dry blot technique, applying 323 mA current for 30 minutes. The membrane was blocked for one hour using 5% milk diluted in TBS (0.3% Tween) while shaking. Anti α-syn antibody, diluted 1∶1,000 (Santa Cruz Biotechnology) in 5% milk in TBS (0.3% Tween), was added to the membrane for two hours of incubation followed by several washes with TBS (0.3% Tween). Rabbit anti-mouse IgG (Fc specific)-HRP-conjugated antibody diluted 1∶5,000 in 5% milk diluted in TBS (0.3% Tween) was added for one hour at room temperature while shaking. Blots were developed after thorough TBS (0.3% Tween) washes, using an Enhanced Chemiluminescence System (ECL) according to the manufacturer's manual.

### NMR analysis

Peptide samples were prepared from peptides stored in lyophilized form, dissolved in a solution containing 10% deuterium oxide in 20 mM phosphate buffer and 50 mM NaCl in ddH_2_O. Samples of peptides complexed with α-syn were prepared from stock solutions of α-syn in 20 mM phosphate solution to which NaCl and deuterium oxide were added to achieve the above-mentioned concentrations, and the lyophilized peptide was added at the designated molar ratio. pH was measured for all samples. Samples were prepared in Shigemi tubes with a final volume of 260 µl.

The NMR experiments were performed on a Bruker Avance 600 MHz DMX spectrometer operating at the proton frequency of 600.13 MHz using a 5 mm selective probe equipped with a self-shielded xyz-gradient coil. The transmitter frequency was set on the HDO signal, which was calibrated according to temperature (4°C–4.974 ppm; 10°C–4.821 ppm; 15°C–4.773 ppm; 37°C–4.658 ppm). TOSCY [35], [36] and NOESY [36], [37] experiments were acquired for each temperature and sample set.

Spectra were processed and analyzed with TOPSPIN software (Bruker Analytische Messtechnik GmbH). Zero filling in the indirect dimension, and data apodization with a shifted squared sine bell window functions in both dimensions, were applied prior to Fourier transformation for optimal resolution. The baseline was further corrected in the direct dimension with a quadratic polynomial function.

Resonance assignment was done according to the sequential assignment methodology developed by Wüthrich [John Wiley & Sons, 38] based on the TOCSY and NOESY spectra measured under identical experimental conditions. Chemical shift deviations of the HN-Hα peaks were read from carefully calibrated, highly resolved, strongly apodized overlaid 2D spectra.

### *In vitro* assay of peptide stability

Peptides were dissolved to give a 1 mM solution in 50 µM Tris buffer (pH 7.6). 120 µl of the peptide solution was diluted into a 10% freshly-made homogenate of mouse brain excluding the cerebellum (in 1× Tris buffer and 0.5% Triton X-100). A mixture containing 20% peptide solution and 80% mouse brain homogenate was incubated at 37°C with delicate shaking for two hours. The enzyme reaction was stopped by adding 0.1 M HCl solution, followed by denaturation of the protein using methanol and incubation at 20°C for one hour. The precipitated proteins were centrifuged at 29,000×g for 20 minutes at 4°C and the supernatant containing the peptide was concentrated under vacuum and separated using a C18 HPLC column. The area of the peak (UV absorbance at 280 nm) corresponding to the intact peptide was compared with an equivalent sample incubated in 50 µM Tris buffer.

### Cell line

SH-SY5Y cells (kindly provided by Prof. Daniel Offen, Tel Aviv University), stably transfected with wild type α-syn, were maintained with DMEM: F12 1∶1 containing 5% fetal bovine serum, 2 mM L-glutamine, 1000 U/ml penicillin-G sodium, 1 mg/ml streptomycin sulfate and 1 mM sodium pyruvate under selective conditions with 100 µM G-418 at 37°C with 5% CO_2_. Cells underwent differentiation with 10 µM retinoic acid (Sigma) in complete growth medium, replaced every two days for a period of eight days.

### Peptide internalization into SH-SY5Y cells

The retro-inverso β-syn 36 peptide internalization into SH-SY5Y cells was visualized by immunocytochemistry staining. 10^4^ cells were seeded on a cover slip coated with poly-L-lysine (0.1%) in a 24-well plate and underwent differentiation as described. Differentiated cells were incubated with the peptide in the cells growth medium for 30 minutes to 4 hours at 37°C. Cells were washed with PBS and fixed with 4% paraformaldehyde in PBS for 30 minutes at room temperature and then washed twice with PBS and permeabilized with 0.1% Triton in PBS for 2 minutes. Following two PBS washes, cells were blocked with 10% normal goat serum in 3% BSA for 30 minutes and incubated with anti α-syn antibody (Santa Cruz Biotechnology) diluted 1∶1000 and Phalloidin 4 µg/ml (Sigma) for one hour, followed by an additional hour of incubation with Cy5-conjugated goat anti-rabbit IgG (Jackson ImmunoResearch). After being thoroughly washed with PBS, cells were mounted using ProLong Antifade (Invitrogene). Images were taken with LSM510 confocal microscope (Zeiss).

### Fly keeping

Flies were reared on standard cornmeal-molasses medium and were kept at 25°C. As Drosophila females can store sperm cells in their bodies, crosses were conducted using virgin females collected no longer than eight hours after eclosion at 25°C or 18 hours after eclosion at 18°C. The crosses were performed at 29°C. Adult offspring (F1) from the crosses were collected up to 9 days after the beginning of their eclosion at 25°C in order to avoid offspring from the next generation (F2).

### Fly crossing

Male flies carrying the driver *elav^c155^*-Gal4 on their X chromosome were crossed to females carrying the UAS-regulated A53T α-syn transgene located on their X chromosome (kindly provided by Prof. Mel Feany, Harvard Medical School). This resulted in first generation (F1) female offspring expressing A53T α-syn in their nervous system which served as our PD fly model. In parallel, male flies carrying the driver *elav^c155^*-Gal4 on their X chromosome were crossed to wild type (Oregon-R) females and the resultant F1, carrying only the *elav^c155^*-Gal4 driver served as control

### Special fly feeding

The retro-inverso β-syn 36 peptide was added to standard cornmeal-molasses medium, upon cooking, at a concentration of 0.75 mg/ml. The peptide was mixed thoroughly into the medium when it was at 40°C and the mixture was aliquoted into fly rearing vials. The vials were kept at 4°C until use. Crosses were performed either on regular Drosophila medium (control) or on medium supplemented with the peptide. Animals fed on the appropriate medium from the beginning of the larval stage. After eclosion, adult offspring were transferred into a fresh vial containing regular Drosophila medium on top of which a solution of 0.75 mg/ml peptide was dripped every other day.

### Locomotive (climbing) assay

Vials of each of the following four F1 classes: 1) Females expressing A53T α-syn reared on regular medium; 2) Females expressing A53T α-syn reared on medium supplemented with the tested compound; 3) Control females carrying only the *elav^c155^*-Gal4 driver, reared on regular medium; 4) Control females carrying only the *elav^c155^*-Gal4 driver, reared on medium supplemented with the tested compound, each containing 10 flies were tapped gently on the table and were let to stand for 20 seconds. The percentage of flies which climbed along the test tube was monitored over time. Each class had four independent vial repeats.

### Statistical analysis

P-values were calculated for comparison of female flies expressing A53T α-syn reared on regular medium with female flies expressing A53T α-syn on medium supplemented with the tested compound and with control flies using one tail ANOVA test with a value of 0.05. P<0.05 was considered significant.

### Immuno-staining of adult fly brains

Fourteen-day old adult flies were dissected and their brains were removed. After incubation in Paraformaldehyde the whole brains were transferred to formic acid and blocked with 0.5% Triton in 5% BSA. Following an overnight incubation with anti α-syn antibody (Santa Cruz Biotechnology) diluted 1∶200, the brains were stained with Cy5-conjugated goat anti-rabbit IgG (Jackson ImmunoResearch). After being thoroughly washed with PBS, cells were mounted using ProLong Antifade (Invitrogene). Images were taken with LSM510 confocal microscope (Zeiss).

## Results

### Identification of the recognition domains between β-syn and α-syn

**Figure 1:**
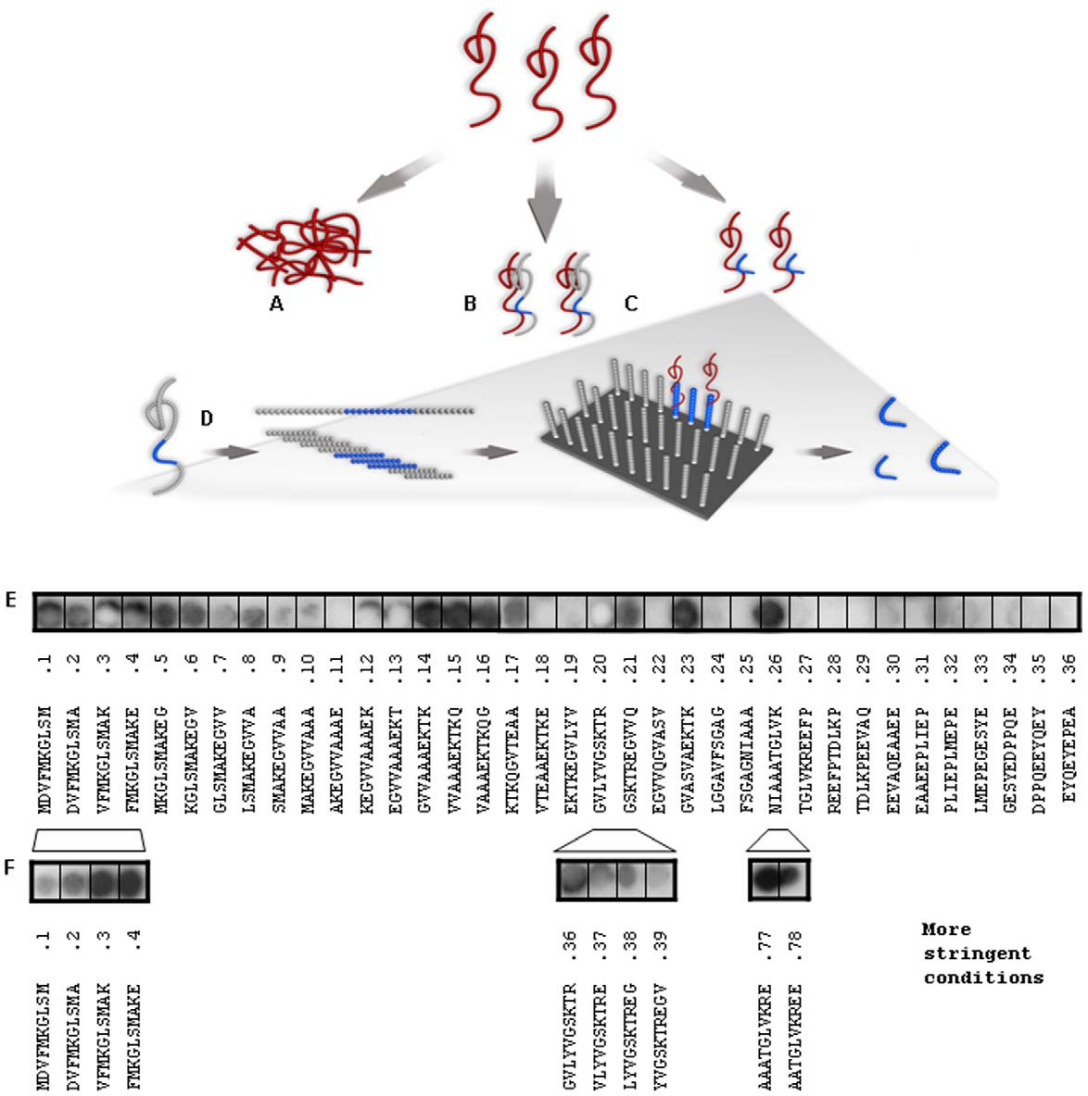
Mapping of the interaction areas between β-syn and α-syn. (A) Schematic illustration of interactions between β-syn and α-syn; α-syn monomers (red) aggregate to form fibrils and oligomers. (B) β-syn (grey-blue) is known to inhibit the aggregation of α-syn. (C). Potential β-syn-derived peptides (blue) as inhibitors of α-syn aggregation. (D) Molecular mapping of β-syn sequences that bind α-syn using peptide-array analysis. (E) Screen for α-syn binding sequences within the β-syn protein. Decamer peptides corresponding to consecutive 9 residue overlapping sequences whithin the first 15 amino acids of β-syn and 5 residue overlapping sequences of the rest of the protein were synthesized on a cellulose membrane and incubated with His-labeled α-syn. (F) An additional screen was performed using more stringent conditions and a 9 residue overlapping along the entire protein.

The full length β-syn has been shown to inhibit the aggregation of α-syn in a dose-dependent manner. Therefore the recognition modules within the β-syn protein were systematically mapped (illustrated in Figure 1A–D).

An overlapping peptide array technique was used for fine molecular mapping using 9 amino acids overlapping decamer peptides for the first 15 amino acids of the protein and 5 amino acids overlapping decamer peptides for the rest of the protein (Figure 1E). For validation, an additional set of decamer peptides overlapping in 9 amino acids were synthesized on a membrane and their binding detection to α-syn was performed under more stringent conditions. A few binding regions were detected in the first screen but only some of them appeared under more stringent binding conditions (Figure 1F). These results lead to the synthesis of seven candidate decamers for further analysis. These peptides were termed β-syn 6, 14, 36, 37, 38, 39, 77, and 78 according to the location of their first amino acid (Table 1). A septamer, containing amino acids 9–15 termed β-syn 6short (Table 1), that was shown to display significant inhibition of α-syn aggregation [32] was used as positive control.

**Table 1:** β-syn derived peptides.

Peptide name	Sequence	Position within β-syn protein sequence
β-syn 6short (control)	SMAKEGV	9–15
β-syn 14	GVVAAAEKTK	14–23
β-syn 36	GVLYVGSKTR	36–45
β-syn 37	VLYVGSKTRE	37–46
β-syn 38	LYVGSKTREG	38–47
β-syn 39	YVGSKTREGV	39–48
β-syn 77	AAATGLVKRE	77–86
β-syn 78	AATGLVKREE	78–87
**β-syn 36 modifications (all aa are D enantiomers)**		
β-syn 36D	GVLYVGSKTR	36–45
Retro inverso β-syn 36	RTKSGVYLVG	36–45
Acetylated and amidated retro inverso β-syn 36	Acetyl-RTKSGVYLVG-amide	36–45
β-syn 36D short	YVGSKTR	39–45
Y39W β-syn 36D	GVLWVGSKTR	36–45

The peptides containing amino acids 36–39 of β-syn were of special interest due to the presence of tyrosine. We and others have previously identified an important role of aromatic amino acids in the acceleration of amyloid fibrils formation [5], [6], [9], [39], [40]. Therefore, the presence of tyrosine within these peptides might be useful in the inhibition of the self-assembly of α-syn.

### Inhibition of formation of α-syn amyloid fibrils by β-syn-derived peptides

**Figure 2:**
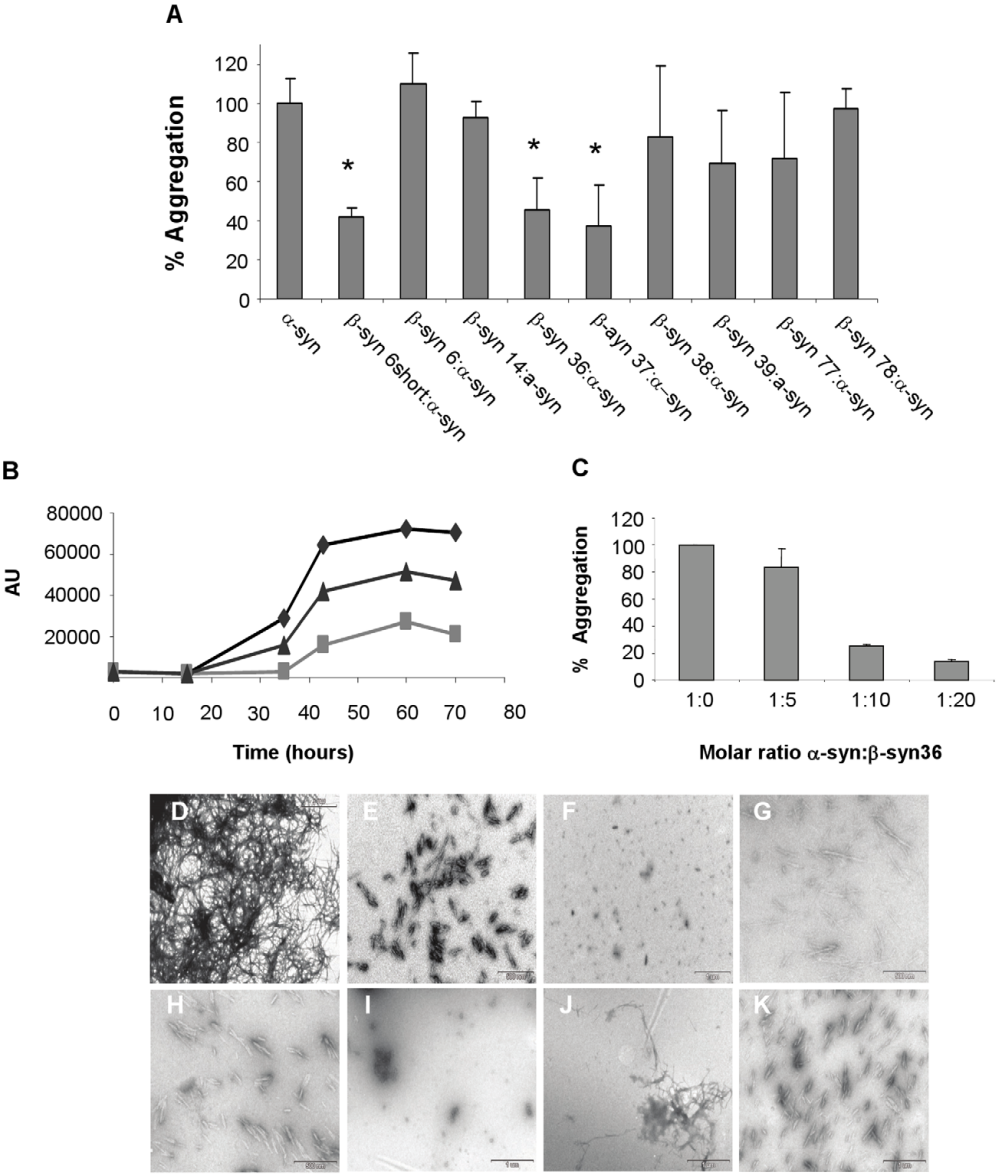
*In vitro* analysis of α-syn aggregation. (A) *In vitro* inhibition of α-syn fibrillar assembly. β-syn peptides were screened for inhibiting α-syn aggregation using ThT (molar ratio 20∶1 respectively). (B) Kinetic analysis of the aggregation of α-syn in the presence of β-syn peptides 36 and 39. Control α-syn (♦), peptide β-syn 39 with α-syn (▴), peptide β-syn 36 with α-syn (▪). (C) Dose dependent inhibition effect of peptide β-syn 36 on α-syn aggregation. (D-K) TEM images of α-syn fibrils with several peptides; α-syn alone, α-syn with β-syn 6short, 36, 37, 38, 39, 77, 78. 6 short, 37 and 38; bar = 500 nM. Rest of the peptides bar  =  1 µM. ^*^, P<0.08.

Thioflavin-T (ThT) binding assay was used to examine and quantify the inhibitory effect of the β-syn peptides on α-syn fibril formation. This method provides quantitative information on amyloid fibril growth. α-syn was incubated for several days at 37°C with vigorous shaking to allow the formation of amyloid fibrils with or without the different β-syn-derived peptides. The fibrillization process was monitored for three days. Figure 2A demonstrates that the formation of amyloid fibrils was significantly reduced in the presence of several of the peptide inhibitors. A kinetic analysis was performed for the two potential inhibitors β-syn 36 and β-syn 39 (Figure 2B) as these two peptides represent the first and last peptides screened within the aromatic region (Table 1). β-syn 36 showed better inhibition than β-syn 39 and dose-dependent inhibition tests revealed that β-syn 36 is still efficient at an excess of 10∶1 (β-syn 36: α-syn) molar ratio but is less efficient at of 5∶1 ratio (Figure 2C).

### TEM analysis of α-syn aggregation

Transmission electron microscopy (TEM) analysis was performed on samples of α-syn incubated with and without the β-syn inhibitor peptides. The samples were taken from the ThT experiment (presented in Figure 2A). The t-test showed P<0.08.

While the fibrils formed by α-syn alone were large, broad and had a ribbon-like shape (Figure 2D), only short or no fibrils were detected in the presence of the β-syn peptides. β-syn 36 almost completely inhibited fibril formation (Figure 2F). These results are highly correlated with the results of the ThT assay.

### Screening for inhibition of α-syn oligomer formation

**Figure 3:**
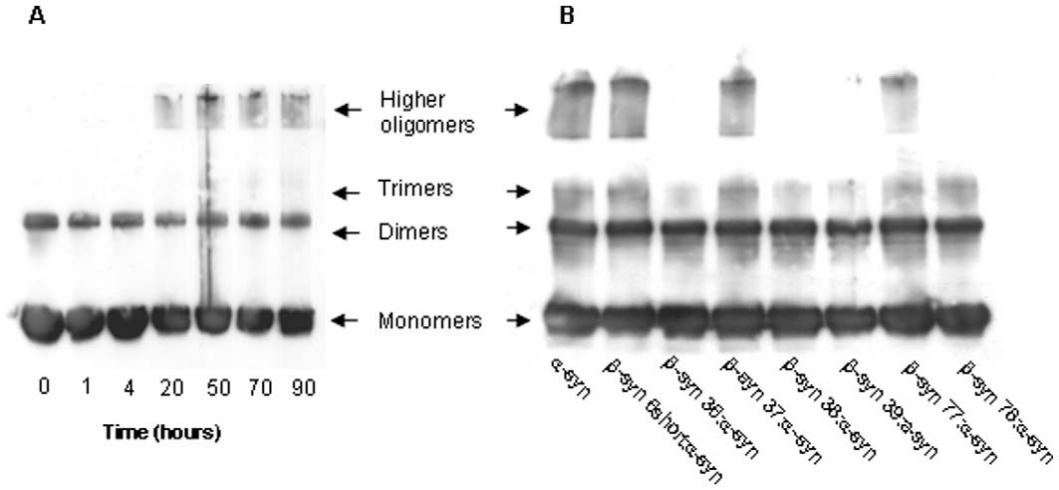
(A) Aggregation of α-syn over time. Soluble oligomers were formed while shaking at 37°C. Detection was carried using western blot analysis. (B) Inhibition of oligomer assembly using β-syn peptides following 24 hours of incubation.

To examine the ability of the peptides to inhibit the early stage of α-syn aggregation, soluble fractions of α-syn were collected after incubation with and without the β-syn peptides. The reaction mixtures were separated by SDS-PAGE followed by western blot analysis using a specific anti α-syn antibody (Santa-Cruz). A preliminary test of α-syn oligomer formation over time in the absence of β-syn-derived peptides revealed that high oligomers could be detected after ∼20 hours of incubation (Figure 3A). Therefore samples incubated with β-syn-derived peptides were collected after this period of time. Given the current debate regarding the identity of the toxic species in the process of α-syn aggregation, it was interesting to note that some β-syn-derived peptides affected the formation of α-syn fibrils while others affected the formation of oligomers (Figure 3B). The β-syn 6short peptide (our positive control peptide) and the β-syn 37 peptide inhibited formation of mature fibrils but not oligomers, while the β-syn 78 peptide inhibited oligomers but not fibril formation. The β-syn 36 peptide inhibited almost completely the formation of both aggregation types (Figure 2F, 3B).

### Modified versions of β-syn 36 peptide inhibit the aggregation of α-syn and have increased serum stability

**Figure 4:**
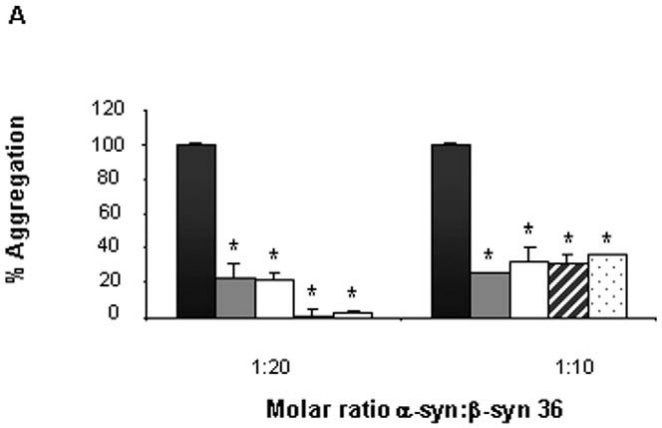
Inhibition of α-syn fibrillar assemblies using ThT fluorescence assay. Three modified peptides of β-syn 36 were screened for their inhibition on α-syn aggregation: α-syn alone (black), β-syn 36 with α-syn (grey), and the modified peptides with α-syn: β-syn 36D (white), retro-inverso β-syn 36 (cross-hatch), acetylated and amidated retro inverso β-syn 36 (dots). The molar ratios of α-syn:peptide were 1∶20 and 1∶10. *, P<0.08.

**Figure 5:**
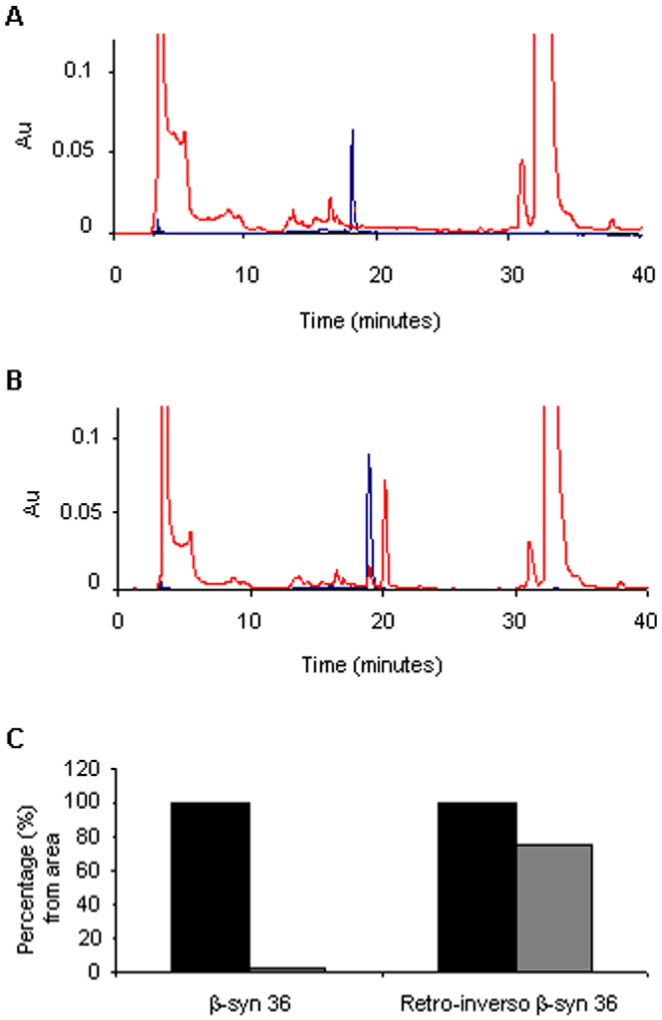
Serum stability of β-syn 36 peptides. Brain extract of wild type ICR white mouse was incubated with the peptides β-syn 36 and retro-inverso β-syn 36 for two hours and loaded on a C18 column for serum stability analysis. (A) Spectra of the β-syn 36 peptide (blue) and β-syn 36 peptide with brain extract (red). (B) Spectra of the retro-inverso β-syn 36 peptide (blue) and retro-inverso β-syn 36 peptide with brain extract (red). (C) Quantification of the area under the peak. The area of the peptide in time ‘0’ was determined as 100%. The left columns represent the peptide (black) and the right columns represent the peptide with the brain extract (grey).

For using the β-syn 36 peptide as potential drug it is desirable to increase its half-life in the patient's serum, by reducing its degradation by tissue and serum proteases and peptidases. β-syn 36 is a decamer peptide comprising natural L-amino acids hence is susceptible to proteolytic degradation. Three modified, more stable versions of β-syn 36 peptide were designed: the first composed of D instead of L-amino acids, termed β-syn 36D; the second was an analogue with retro-inversion of the amino acids sequence, composed of D-amino acids assembled in the reverse order from that of the parent L-sequence, termed retro-inverso β-syn 36. To negate the terminal negative and positive charges of the retro-inverso peptide, a third modified N-terminal acetylated and C-terminal amidated peptide was synthesized, termed acetylated and amidated retro-inverso β-syn 36. The modifications of these peptides were suspected to alter the efficacy of the lead peptide without harming its biological activity. Indeed, when tested for their ability to inhibit α-syn fibril formation, using the ThT binding assay with a molar ratio of 1∶20 or 1∶10 in favor of the peptides, all three modified versions demonstrated a similar biological effect as β-syn 36 (Figure 4). The t-test showed P<0.08. Next, the serum stability of the unmodified β-syn 36 peptide and the modified retro-inverso peptide was compared by incubating them for two hours in mouse brain homogenate to model serum degradation. The results showed that the modified peptide is more stable than the unmodified β-syn 36 (Figure 5).

To evaluate the binding of the peptides to α-syn, increasing amounts of α-syn monomers were titrated into a solution of the modified β-syn 36 peptide and anisotropy was determined (Figure S1). The affinity constant was determined to be ∼1 µM.

### NMR analysis of the interaction of β-syn peptides with α-syn

Studying interactions between α-syn and β-syn by NMR is severely limited by signal overlap due to the similarity in their sequences. This was overcome by using the all-D chirality retro-inverso β-syn 36 peptide (retro-inverso β-syn 36: R_45_T_44_K_43_S_42_G_41_V_40_Y_39_L_38_V_37_G_36_), which gives a resolved spectrum that is distinct from that of α-syn. The residues that participate in the intermolecular interactions of the retro-inverso β-syn 36 with α-syn were determined by following changes in their amide and Hα chemical shifts upon binding. The spectrum of the peptide in the absence and in the presence of α-syn was determined under several sets of conditions: Molar ratios of 1∶1 and 1∶5 peptide to α-syn; peptide concentrations of 160 and 400 µM; temperatures of 4°C, 10°C, 20°C, 25°C, and 37°C; and using Y39W β-syn 36D analog.

**Figure 6:**
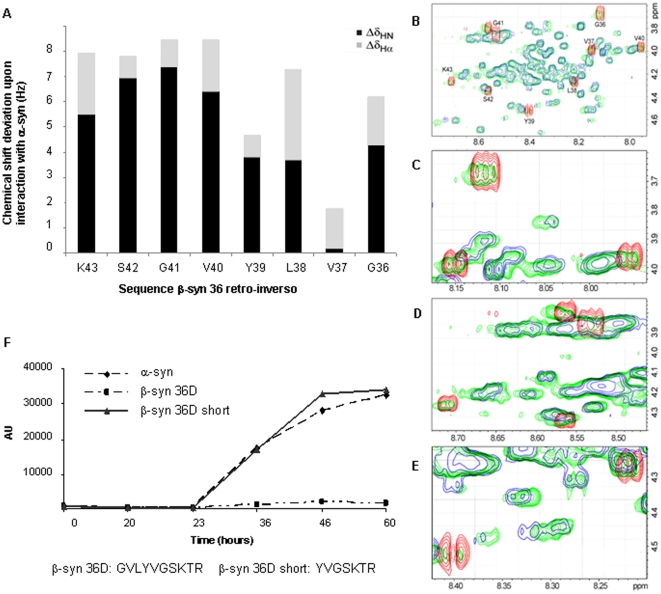
NMR analysis. (A) Chemical shift deviations of HN (black) and Hα (grey) backbone atoms of the β-syn 36 retro-inverso peptide upon binding α-syn. (B) Hα-HN region of TOCSY spectra overlay of the β-syn 36 retro-inverso peptide (red) and α-syn (blue) upon binding (green), (C–E) Expansions of all the retro-inverso peaks that showed deviations upon binding. (F) ThT analysis of fibril formation by α-syn with and without β-syn 36D and its truncated version which lacks amino acids G_36_, V_37_ and L_38_.

All residues of the retro-inverso β-syn 36 peptide were identified and assigned (Figure S2). R_45_ and T_44_ were not detected in the amide region, but were identified in the aliphatic region. In all samples some of the N-terminal region amide signals were lost between G_41_ and R_45_; however, all samples showed the general trend found for the sample of retro-inverso β-syn 36 with α-syn at a 1∶1 molar ratio, 160 µM, 4°C, at pH 6.7 (Figure 6A and B-E and Table 1). The amide resonances showed stronger HN and Hα deviations in residues K_43_, V_40_, L_38_ and G_36_. The participation of L_38_ and G_36_ in binding was ascertained using an additional truncated peptide, β-syn 36D short, which lacks the first three amino acids, G_36_, V_37_ and L_38_, and showed no inhibition of α-syn aggregation in ThT assay (Figure 6F). These results suggest that K_43_, V_40_, L_38_ and G_36_ participate in the binding interaction and that the large chemical shift deviation of L_38_ and G_36_ is not only due to their position at the peptide terminus.

### Internalization of β-syn-derived peptides into mammalian cells

**Figure 7:**
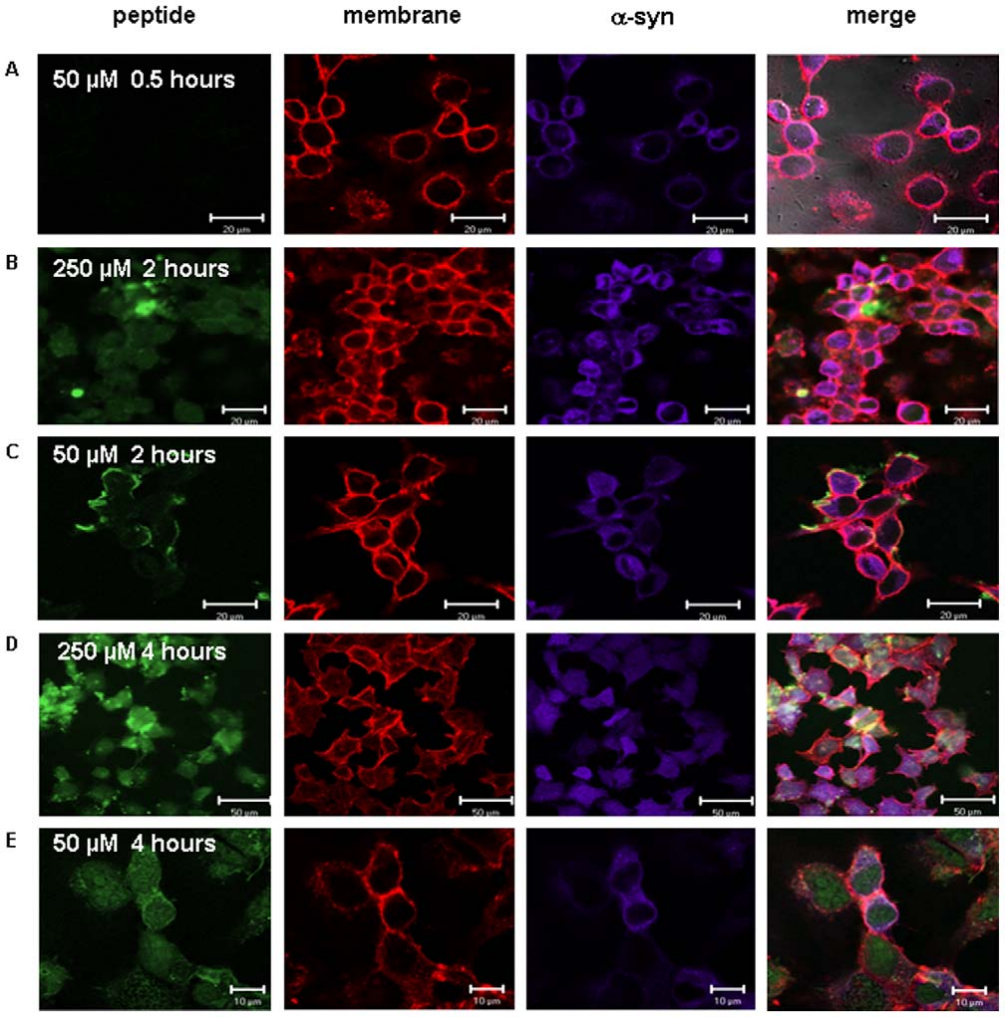
Internalization of retro-inverso β-syn 36 into the cells. Differentiated SH-SY5Y cells over expressing wild type α-syn were incubated with 50 µM and 250 µM of FITC-conjugated retro-inverso β-syn 36 peptide for periods of half an hour, two hours and four hours. After fixation, the presence of the FITC-conjugated peptide (green) was monitored inside the cells. Cellular α-syn was detected using cy5-conjugated antibody (purple) and the cell membrane was marked with Phalloidin (red). (A) After 30 minutes at 37°C there was no peptide staining (green). (B–C) After two hours of incubation, no or little amount of peptide was detected inside the cells (green). (D–E) After 4 hours of incubation, the peptide was clearly detected inside the cells. Peptide localization was visualized using an LSM-510 Zeiss confocal microscope.

To evaluate the efficacy of the β-syn-derived peptide in mammalian cell culture, which serves as a model for PD, SH-SY5Y5 cells over-expressing wild type α-syn were used. They were first subjected to retinoic acid treatment to induce cell differentiation [41]. After eight days of treatment, cells were cultured into fresh growing media for 0.5–4 hours followed by incubation with FITC-conjugated retro-inverso β-syn 36 peptide (green fluorescence). After incubation, cells were washed, fixed, and permeabilized. Overexpression of wild type α-syn in these cells was verified using cy5-conjugated goat anti-rabbit antibody (purple fluorescence) and the cell membrane was marked using Phalloidin reagent (red fluorescence) (Figure 7). The two concentrations of β-syn-derived peptide, 50 µM and 250 µM, showed similar results. After 30 minutes of incubation the peptide did not enter into the cells. No attachment to the cells was noted. After two hours of incubation the peptide was detected mostly on the cell surface. Small amount was detected inside the cells at the concentration of 250 µM. After four hours of incubation, the peptide was clearly visible inside the cells (Figure 7).

### The effect of the retro-inverso β-syn 36 peptide *in vivo*

**Figure 8:**
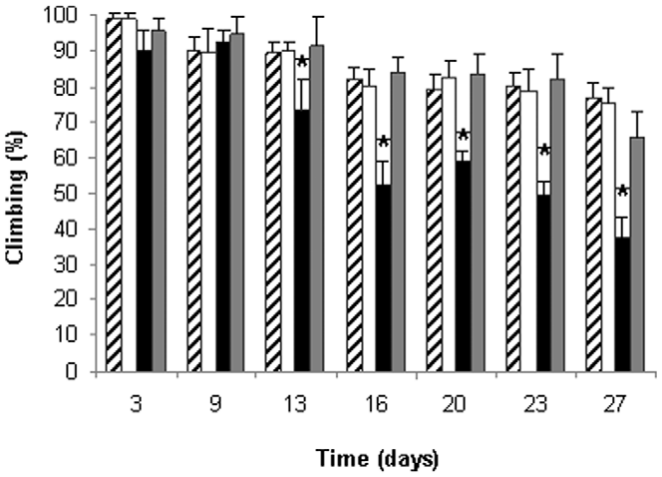
Effect of β-syn-derived peptide on locomotive behavior of PD model flies. Four classes of flies, each containing five tubes of ten flies were analyzed using the climbing assay: Flies expressing α-syn A53T grown on a regular medium (black); flies expressing α-syn A53T grown on medium containing 0.75 mg/mL β-syn retro inverso peptide (grey); Control flies, not expressing α-syn A53T, grown on a regular medium (cross-hatch); Control flies, not expressing α-syn A53T, grown on medium containing 0.75 mg/mL β-syn retro inverso peptide (white). Results show the percent of flies which climbed along the test tube after 20 seconds. ^*^, P<0.05.

The effect of the retro-inverso β-syn 36 peptide on α-syn aggregation in the living organism was assessed using a Drosophila model of PD. These transgenic flies over- express the mutated A53P α-syn in their nervous system, via the Gal4-UAS system. A common behavioral phenotype of these flies is age-dependent defective locomotion: While normal flies tend to climb up the side of the rearing tube, A53T α-syn-expressing flies remain at the bottom [42]. Crossing male flies carrying the pan-neuronal elav-Gal4 driver (on their X chromosome) to females carrying the UAS-regulated A53T α-syn transgene resulted in female offspring expressing A53T α-syn in their nervous system. This cross was performed either on regular Drosophila medium or on medium supplemented with 0.75 mg/mL of the retro-inverso β-syn 36 peptide. The climbing ability of the flies was monitored for 27 days (Figure 8). Locomotion of flies expressing A53T α-syn in their nervous system deteriorated significantly from day 13 onwards. The untreated flies became almost immobile by day 27, while the control classes were very active at this time. In contrast, the treated flies displayed dramatic improvement, behaving almost identical to the control classes, presenting an increase of nearly 86% in their climbing ability in comparison with the untreated group. The peptide had no significant effect on locomotion of the control flies. These results indicate significant phenotypic recovery of the A53T α-syn flies by the retro-inverso β-syn 36 peptide. One tail ANOVA statistics showed P<0.05.

**Figure 9:**
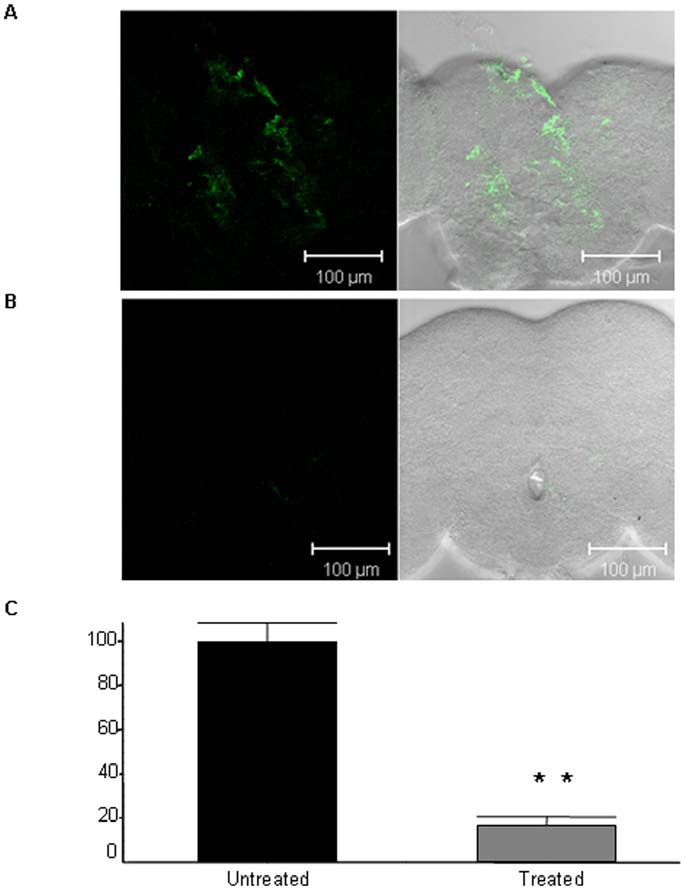
Immunostaining of PD model flies brains. Brains of fourteen-day flies were extracted and immunostained with anti α-syn antibody followed by a Cy5-conjugated antibody. α-syn is detected in green. (A) Staining of untreated flies expressing α-syn A53T grown on a regular medium. (B) Staining of flies expressing α-syn A53T treated with β-syn retro inverso peptide. (C) Quantification of treated and untreated brains. The t-test analysis showed **, P<0.0001.

To evaluate the effect of the retro-inverso β-syn 36 peptide on α-syn accumulation in the brains of these flies, adult flies were fed or unfed with the peptide, and their brains were immunostained with anti α-syn antibody. While a massive accumulation of α-syn aggregates was detected in the brains of the untreated flies (Figure 9A), the brains of the treated flies exhibited a greatly reduced α-syn staining (Figure 9B). Quantification of 8 treated brains and 7 untreated brains was performed and t-test analysis showed **, P<0.0001 (Figure 9C).

## Discussion

The pathological hallmark of PD is the occurrence of insoluble intracellular inclusions termed LB and LN within the surviving dopaminergic neurons of the *substantia nigra* and several other brain regions, with α-syn as the major component of these inclusions [21]. The first indication that α-syn could be involved in neurodegenerative diseases came from work on the NAC region isolated from brain amyloid preparations of AD patients [43]. Two different point mutations, resulting in amino acid substitutions A53T and A30P, have firmly established the pathogenic importance of α-syn when they were indentified as a cause of rare, inherited forms of PD [19]. Although the etiology of idiopathic PD is unknown, it is likely to be multifactorial. A presumptive feature leading to PD is the aggregation of α-syn, which can be triggered by a range of factors, both environmental and endogenous [31]. Uversky et al. suggested that the cellular levels of β-syn and γ-syn could constitute such an endogenous factor [31].

Due to the importance of α-syn aggregation in the pathology of PD, a useful strategy in drug development is to design short peptides, or small molecules, that can block, slow down, or reverse α-syn aggregation, particularly at its early stages [27]. The fact that α-syn is a natively unfolded protein, makes the rational design of compounds that can stabilize the native, nontoxic conformation of α-syn a challenging task [27].

A recently proposed approach is to use the properties of the β-syn protein as a natural inhibitor of the aggregation of α-syn [32]. In the present work we applied this approach and for the first time systematically mapped the entire sequence of β-syn to identify all the domains that have the potential to mediate molecular recognition events between β-syn and α-syn. Using the peptide array technique several interaction modules were identified and some of them were verified by a second round under more stringent interaction conditions. Decamer peptides corresponding to the interaction regions were synthesized and their ability to inhibit the aggregation of α-syn was tested using ThT and TEM assays. Both methods showed consistent results, indicating an inhibitory effect of the peptides on fibril formation by α-syn.

Recent studies reveal that early “soluble oligomers” rather than mature amyloid fibrils are likely the pathogenic species that drive neurodegeneration and neuronal cell death [44], [45]. It is believed that the conversion of α-syn into these oligomers, and then into insoluble fibrils, is initiated by conformational changes from random coil to β-sheet structure [4], [27], [46], [47]. This suggests that a candidate anti-amyloid compound should block the very early stages of amyloid oligomerization. Consequently, we tested the ability of the interacting β-syn-derived peptides to inhibit the formation of the soluble fraction of α-syn, and found several peptides that were able to do so, while the positive control peptide appeared to inhibit only the formation of the insoluble fibrils.

While several peptides such as β-syn 6short and β-syn 37 inhibit fibril formation but not oligomer formation, β-syn 78 inhibits oligomer formation but not fibril formation. These results support the latest theory of a two-pathway mechanism of amyloids aggregation. A similar hypothesis was demonstrated on β-amyloid in AD [48].

Our best inhibitor candidate was the β-syn 36 peptide which inhibited both the soluble and insoluble fibrillar aggregates of α-syn. In addition to its inhibitory properties, this peptide showed no toxicity towards cultured cells (PC12) when added, at concentrations up to 1 mM, to the their growing medium and incubated for twenty four hours (data not shown).

The sequence of the β-syn 36 peptide has two interesting features. First, it contains part of the repetitive domain of KTKEGV (with a substitution of K for R), which has been suggested to represent an evolutionary balance between the functional conformer of α-syn (α-helix and/or random coil) and its pathogenic β-sheet conformation [49]. Second, the β-syn 36 peptide contains the aromatic amino acid tyrosine, which according to the “aromatic stacking” hypothesis previously suggested by us [6], may contribute to the binding energetics as well as induce order and directionality to the self-assembly of amyloid structures. These two characteristics may play a role in the inhibitory potential of this peptide.

Two problems commonly associated with peptides in therapy are their high sensitivity to proteolytic degradation and lack of permeation into the cells [50]. We attempted to overcome the first obstacle by using modified peptides containing D amino acids and by reversing the peptide sequence. Both modifications retained the inhibitory effect of the peptides. In addition to its excellent inhibition ability, the retro-inverso β-syn 36 modified peptide displayed increased stability towards proteolytic degradation. Concerning the cell permeability, we showed that the stable retro-inverso β-syn 36 peptide can penetrate differentiated SHSY5Y cells without adding any internalization motif.

In this context, it is worth mentioning that although α-syn is a typical cytoplasmic protein, a small amount of both monomeric and aggregated forms were reported to be secreted from cells and exist in human body fluids, such as cerebrospinal fluid [51], [52]. Furthermore, extracellular α-syn was shown to be capable of entering cells, and the mechanism of its internalization was suggested to depend on the assembly state of the protein: aggregated forms of α-syn, both fibrils and oligomers, enter into cells via receptor-mediated endocytosis, while monomeric α-syn diffuses passively across the plasma membrane [53]. It is worth noting that there is high homology between α-syn and β-syn, especially in the N terminal region, and the sequence of the β-syn 36 peptide can be detected within the sequence of α-syn at the same position with one amino acid substitution of lysine to arginine. We therefore speculate that the identified peptide either diffuses passively across the plasma membrane or enters into cells via the same receptor-mediated endocytosis as α-syn. In accordance with the *in vitro* results, the retro-inverso β-syn 36 modified peptide alleviated α-syn engendered symptoms in a transgenic fly model of PD and significantly reduced the accumulation of aggregated α-syn in their brains. Despite its specificity to α-syn, it will be interesting to examine the efficacy of the peptide in other fly models of amyloidogenic proteins such as polyQ and β-amyloid.

The backbone chemical shift deviations of the retro-inverso β-syn 36 peptide upon binding to α-syn, as determined by NMR, are largest for the hydrophobic residues L_38_ and V_40_, in addition to the more flexible terminal residues K_43_ and G_36_. Residues L_38_ and V_40_ surround the aromatic Y_39_, previously shown to participate in the oligomeric interaction [5], [6], although Y_39_ itself showed little change in chemical shift upon binding α-syn. It is possible that the bulky side chain of Y_39_ stabilized a local conformation in the unbound structure, which was not significantly changed upon interacting with α-syn. The two neighboring hydrophobic residues, L_38_ and V_40_, may reside within a hydrophobic binding interface with α-syn. The chemical environment of this region, reflected in the deviations in chemical shift, changes from a monomeric, exposed conformation to a more shielded, hydrophobic environment upon interacting with α-syn.

Taken together, our results support the idea of using the naturally occurring β-syn protein for the development of inhibitors of α-syn aggregation. We have mapped a decamer peptide in β-syn which is able to inhibit both the early and late stages of α-syn aggregation and we were able to stabilize it using several modifications. The modified decamer peptide showed a significant amelioration of PD-associated defects in a transgenic fly model.

## Supporting Information

Figure S1 The affinity of the modified β-syn 36 containing tryptophan instead of tyrosine towardsα-syn monomers was examined using fluorescent anisotropy. Kd  =  1 µM.
(0.02 MB TIF)

Figure S2 NMR assignment spectra of β-syn 36 retro-inverso peptide. Overlay of HN-Hα interaction regions of TOCSY (red) and NOESY (green) spectra of taken under identical conditions according to which assignment was performed.
(8.35 MB TIF)
