# Supplementary figures and images for: Inhibiting α-Synuclein Oligomerization by Stable Cell-Penetrating β-Synuclein Fragments Recovers Phenotype of Parkinson's Disease Model Flies
Source: PLoS One. 2010 Nov 10;5(11):e13863. doi: 10.1371/journal.pone.0013863 (PMC2978097; doi:10.1371/journal.pone.0013863)

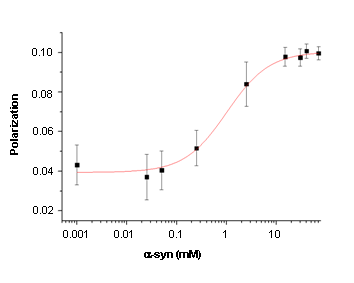

Supplement: Figure S1 — The affinity of the modified β-syn 36 containing tryptophan instead of tyrosine towardsα-syn monomers was examined using fluorescent anisotropy. Kd = 1 µM. (0.02 MB TIF) [file pone.0013863.s001.tif]

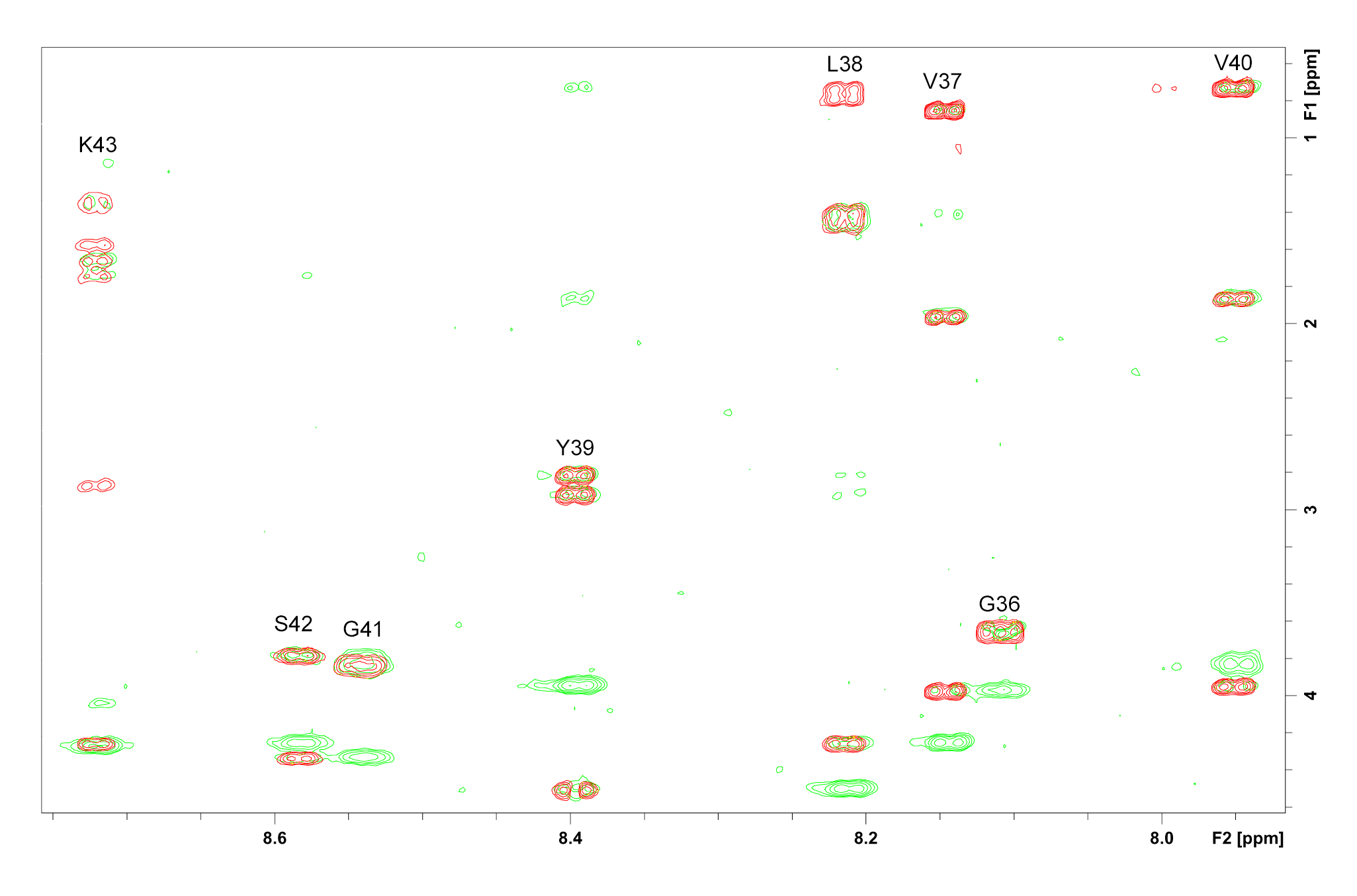

Supplement: Figure S2 — NMR assignment spectra of β-syn 36 retro-inverso peptide. Overlay of HN-Hα interaction regions of TOCSY (red) and NOESY (green) spectra of taken under identical conditions according to which assignment was performed. (8.35 MB TIF) [file pone.0013863.s002.tif]
